# Peptide Vaccines for Leishmaniasis

**DOI:** 10.3389/fimmu.2018.01043

**Published:** 2018-05-11

**Authors:** Rory C. F. De Brito, Jamille M. De O. Cardoso, Levi E. S. Reis, Joao F. Vieira, Fernando A. S. Mathias, Bruno M. Roatt, Rodrigo Dian D. O. Aguiar-Soares, Jeronimo C. Ruiz, Daniela de M. Resende, Alexandre B. Reis

**Affiliations:** ^1^Laboratório de Pesquisas Clínicas, Programa de Pós-graduação em Ciências Farmacêuticas/CiPharma, Escola de Farmácia, Universidade Federal de Ouro Preto, Ouro Preto, Brazil; ^2^Laboratório de Imunopatologia, Núcleo de Pesquisas em Ciências Biológicas, Universidade Federal de Ouro Preto, Ouro Preto, Brazil; ^3^Instituto Nacional de Ciência e Tecnologia em Doenças Tropicais, Salvador, Brazil; ^4^Grupo Informática de Biossistemas e Genômica, Programa de Pós-graduação em Ciências da Saúde, Instituto René Rachou, Fundação Oswaldo Cruz, Belo Horizonte, Brazil; ^5^Programa de Pós-graduação em Biologia Computacional e Sistemas, Instituto Oswaldo Cruz, Fundação Oswaldo Cruz, Rio de Janeiro, Brazil

**Keywords:** peptide-based vaccines, chimeric vaccine, polypeptide vaccines, tegumentary leishmaniases, visceral leishmaniasis

## Abstract

Due to an increase in the incidence of leishmaniases worldwide, the development of new strategies such as prophylactic vaccines to prevent infection and decrease the disease have become a high priority. Classic vaccines against leishmaniases were based on live or attenuated parasites or their subunits. Nevertheless, the use of whole parasite or their subunits for vaccine production has numerous disadvantages. Therefore, the use of *Leishmania* peptides to design more specific vaccines against leishmaniases seems promising. Moreover, peptides have several benefits in comparison with other kinds of antigens, for instance, good stability, absence of potentially damaging materials, antigen low complexity, and low-cost to scale up. By contrast, peptides are poor immunogenic alone, and they need to be delivered correctly. In this context, several approaches described in this review are useful to solve these drawbacks. Approaches, such as, peptides in combination with potent adjuvants, cellular vaccinations, adenovirus, polyepitopes, or DNA vaccines have been used to develop peptide-based vaccines. Recent advancements in peptide vaccine design, chimeric, or polypeptide vaccines and nanovaccines based on particles attached or formulated with antigenic components or peptides have been increasingly employed to drive a specific immune response. In this review, we briefly summarize the old, current, and future stands on peptide-based vaccines, describing the disadvantages and benefits associated with them. We also propose possible approaches to overcome the related weaknesses of synthetic vaccines and suggest future guidelines for their development.

## Introduction

The leishmaniases represent a wide spectrum of parasitic diseases caused by dimorphic protozoan of the genus *Leishmania* (1). The disease has an incidence ranging from 200,000 to 400,00 and from 700,000 to 1 million visceral and cutaneous leishmaniases cases, respectively, occurring each year, and a tentative estimate of 20,000–40,000 leishmaniasis deaths per year. The main clinical forms can be grouped into visceral leishmaniasis, the most severe form of the disease, which can progress to death when untreated; cutaneous leishmaniasis, the most common, which causes ulcerations on the skin; and mucocutaneous leishmaniasis, characterized as a mutilating disease that causes irreversible deformities, mainly of the face (2). In recent decades, *Leishmania* species have spread across the world and reached non-endemic areas (3).

For many decades, the traditional prophylactic strategy concerning vector control using spray insecticides, rodent control using poison baits, environmental management, and control of domestic reservoirs has been used (4, 5). However, none of these strategies were able to effectively decrease the number of canine and human cases (5), and a lack of commitment to preventive campaigns has been reported (6). Thus, development of new strategies for the prevention of the disease has become a high priority (7). In this context, the development of vaccines for leishmaniases becomes a promising tool for prophylaxis in endemic areas, with potential impact on the epidemiology of the disease (8). It is a consensus that Th1 immune response plays a critical role not only in protection against the primary infection but also promoting a lifelong immunity to *Leishmania* re-infection (9). T-cells, namely, CD4^+^ cells, are crucial in immune protection by producing various important cytokines associated with resistance, such as IFN-γ and TNF-α (10). Thus, an ideal vaccine should promote a strong Th1 response against *Leishmania* parasites (11).

An ancient practice of immunization is leishmanization, in which live and virulent *Leishmania* promastigotes are injected in uninfected individuals living in endemic areas. Appearance of severe side effect suggests that leishmanization is unfit for large-scale immunization protocols (12). Regarding whole parasite vaccines, trials in dogs and humans using killed or genetically attenuated parasites. This kind of vaccines offers a huge repertoire of parasite antigens and it can promote significant protection against infection. By contrast, these vaccines display low stability and safety in comparison with other type of vaccines (8, 13–15). Parasite subunits-based vaccines are currently most popular in modern due to their ability to stimulate specific immune response. However, they are not completely safe and they can present side effects (16–19). Despite the existence of various studies in this area, no licensed vaccine is available for humans against any form of leishmaniases (8). Therefore, many different strategies to identify new antigens have been employed to develop a vaccine against leishmaniases (20). In this scenario, peptide-based vaccines are a very attractive alternative because they are based on a short antigenic epitope to trigger a desired immune response. This option may become a promising strategy by promoting not only protection against leishmaniases, but as a potent therapeutic tool to treat the disease (21). Minimal epitopes like peptides are able to elicited strong T-cell-specific responses that are fundamental to eliminate intracellular parasite (22).

This review aims to provide an insightful view over evolution of peptide-based vaccines in leishmaniases prophylaxis, as well as, the most recent innovations in this area.

## Leishmanial Peptide-Based Vaccines

Although many vaccine candidates against leishmaniases are composed of whole parasite or specific proteins, the use of only a minimal pathogen epitope which can stimulate long-lasting protection against the parasite is becoming tendency in vaccine development (22). Peptide-based vaccines are a major focus of this field because they are easier to produce and show more stability than whole attenuated pathogens (22). Furthermore, synthetic peptides have several benefits in comparison with other kinds of antigens, showing an absence of potentially damaging materials, lower antigen complexity, and low costs for scaling up (23). Regarding the immune response, peptide-based vaccines can generate specific responses and they can be combined to design multi-epitopes and/or multi-specific vaccines (24).

Peptide vaccine studies, which were becoming increasingly marginalized just a few years ago, are now on the rise as a promising approach for the rational design of vaccines (22).

In this review, we performed an extensive search for studies involving leishmanial peptide-based vaccines in PubMed and identified 30 original research studies (the methodology applied for the searching and selection the 30 articles is described in statement subtopic) which are demonstrated in Tables 1 and 2.

**Table 1:** Summary of peptides evaluated as potential vaccine candidates against visceral leishmaniasis.

Protein	Species	Epitope (residue)	Finding method	Host organism	Dose/route	Adjuvant	Challenge	Main remarks		Reference
								Immune response	Parasite load or lesion size	
GP63	*Leishmania donovani*	Polytope (561 bp)	*In vitro* assay	BALB/c mice	100 μg/IM	–	2 × 10^7^*L. donovani* promastigotes	↑ IFN-γ and IL-2, ↓ IL-10	↓ Parasite load in spleen and liver	(25)

	*L. donovani*	P1–4 peptides (15–21 aa)	*In silico* prediction (EpiMatrix)	Human PBMC	100 µg	–	–	P1: ↑ IL-10 in PBMCs P4: ↓ IL-10 in PBMCs	–	(26)

KMP-11	*L. donovani*	84 peptides (8 aa)	*In vitro* epitope binding assay	Human PBMC	44 µg mL^−1^ to pulse APC	–	–	↑ IFN-γ by CD8^+^	–	(27)

	*Leishmania infantum*	P12–31 peptide (12–31 aa)	*In silico* prediction (NetMHC3.0 and NetMHCII1.0)	BALB/c mice	10 µg mL^−1^/BM-DCs/IV	CpG ODN	1 × 10^7^*L. infantum* promastigotes	↑ IFN-γ, IL-10, and IL-17; ↑ spleen cells proliferation	↓ Parasite load in spleen and liver	(28)

	*L. infantum*	P1 (20 aa)	*In silico* prediction (SYFPEITHI, BIMAS, and NetMHCII1.0)	BALB/c mice	50 μg/SC	CFA and IFA	–	Poorly immunogenic; no proliferative effects and cytokine secretion	–	(29)

A2	*L. donovani*	Four peptides (17–21 aa) peptides	*In silico* prediction (BIMAS and Protscale)	BALB/c mice	5 μM/pulsed splenocytes/IV	–	1 × 10^7^*L. infantum* promastigotes	CD4-2 and CD8 peptides: ↑ IFN-γ by T-cells CD8 peptide: ↑ specific cytotoxicity by CD8^+^ T-cells	–	(30)

NH36	*L. donovani*	F1, F2, and F3 peptides (~100 aa)	Fragmentation of NH36 in 3 antigens	BALB/c mice	100 μg/SC	Saponin	3 × 10^7^*L. infantum* amastigotes	↑ IFN-γ/IL-10 and TNF-α/IL-10 ratio by CD4^+^ and CD8^+^ T-cells	F3: ↓ parasite load in liver	(31)

Phage display library	*L. infantum*	20 phages with peptides (7 aa)	*In vitro* selection (Bio-panning cycles)	BALB/c mice	1 × 10^11^ phages/SC	Saponin	1 × 10^7^*L. infantum* promastigotes	B10 or C01: ↑ IFN-γ, IL-12, and GM-CSF; ↓ IL-10 and IL-4	B10 or C01: ↓ parasite load in liver, spleen, dLN, and BM	(32)

KMP-11, CPA, CPB, TSA, and P74	*Leishmania* spp.	397 peptides (10 aa)	*In silico* analysis (conservation analyses)	BALB/cj mice	0.2, 2, 10, and 20 µg	–	10 × 10^6^*L. donovani* promastigotes	DNA vaccine: ↑ IFN-γ and TNF-α	DNA vaccine: ↓ parasite load in spleen and liver	(24)

Hypothetical protein	*L. infantum*	Two peptides (9 and 17 aa)	*In silico* prediction (BIMAS)	BALB/c mice	25 μg/SC	Saponin	1 × 10^7^*L. infantum* promastigotes	↓ IL-4 and IL-10	P2: ↓ parasite load in the spleen	(33)

3′-Nucleotidase	*L. donovani*	5 peptides (9 aa)	*In silico* prediction (SYFPEITHI, BIMAS, RANKpepProPredI, and NetMHCpan)	Human PBMC	10 µg mL^−1^	–	–	↑ IFN-γ and IL-2; ↑ T-cell proliferation in PBMC culture and CTL activity	–	(34)

*GP63, glycoprotein 63; KMP-11, kinetoplastid membrane protein-11; A2, amastigote virulent factor; NH36, nucleoside hydrolase 36; CPA, cysteine peptidase A; CPB, cysteine proteinase B; TSA, thiol-specific antioxidant; P74, elongation factor 1-alpha; IFA, incomplete Freund’s adjuvant; CFA, complete Freund’s adjuvant; PBMC, peripheral blood mononuclear cell; CpG ODN, CpG oligodeoxynucleotides*.

**Table 2:** Summary of peptides evaluated as potential vaccine candidates against cutaneous leishmaniasis.

Protein	Species	Epitope (residue)	Finding method	Host organism	Dose/route	Adjuvant	Challenge	Main remarks		Reference
								Immune response	Parasite load or lesion size	
GP63	*Leishmania major*	24 peptides (12–35 aa)	*In silico* prediction (AMPHI)	CBA and BALB/C mice	100 μg/SC or IV	*Corynebacterium parvum*	1 × 10^7^*L. major* promastigotes	↑ IL-2 and IFN-γ; ↑ DTH response	P146–171 peptide: ↓ lesion size	(35)

	*L. major*	7 peptides (14 aa)	*In silico* prediction	BALB/c mice	100 μg/SC	8% poloxamer 407	2 × 10^4^*L. major* promastigotes	↑ CD4^+^ subset proliferation	PT3: ↓ lesion size	(36)

	*L. major*	13 peptides (14 aa)	*In silico* prediction	Human PBMC	50 µg mL^−1^	–	–	PT4, PT7, and PT8: ↑ PBMC proliferation; ↑ IFN-γ	–	(37)

	*L. major*	P154 and P467 modified peptides (16 aa)	*In silico* prediction (AMPHI)	CBA mice	50 μg/SC or IP	–	1 × 10^4^*L. major* promastigotes	↑ IFN-γ and IL-2; ↑ GM-CSF	P467 by SC: ↓ lesion size	(38)

	*L. major*	PT3 peptide (16 aa)	*In silico* prediction	BALB/c mice	100 μg/SC	8% poloxamer 407	2 × 10^4^*L. major* promastigotes	–	↓ Lesion size	(39)

	*L. major*	L1 and L2 peptides (16 aa)	*In silico* prediction	BALB/c mice	100 µg mL^−1^/BM-DCs/IV	–	5 × 10^5^*L. major* promastigotes	L1: ↑ IFN-γ and IL-4	↓ Footpad swelling; ↓ parasite load in LN	(40)

	*Leishmania mexicana*/*L. major*	HLA-A2 peptides (9 aa)	*In silico* prediction (SYFPEITHI)	BALB/c and HHDII transgenic mice	100 + 140 µg of helper peptide/IV	IFA	2 × 10^6^*L. mexicana* promastigotes	C2 peptide: ↑ CTL activity and ↑ IFN-γ in HHDII mice; ↑ CTL activity in BALB/c mice	–	(41)

KMP-11	*Leishmania panamensis*	6 overlapping peptides (20 aa)	*In vitro* epitope binding assay	Human PBMC	10 µg mL^−1^ to pulse APCs	–	–	↑ Lymphoproliferation; ↑ IFN-γ by T-cells	–	(42)

Whole proteome	*L. major*	26 peptides (9–10 aa)	*In silico* prediction (SYFPEITHI, BIMAS, ProPred-I, and MAPPP)	BALB/c mice	250 µg per pool/SC	CFA	–	14 of 26 peptides: ↑ IFN-γ by CD4^+^ and CD8^+^ T-cells	–	(43)

CPB	*Leishmania amazonensis*	9 peptides (8–10 aa)	*In silico* prediction (SYFPEITHI, NetChop, and PAProC)	BALB/c and CBA mice	30 µg mL^−1^	–	1 × 10^6^*L. amazonensis* promastigotes	↑ IFN-γ, IL-12, IL-4, and IL-10; blastogenesis in LN cells	–	(44)

LACK	*L. major*	P158–173 peptide (16 aa)	Immunodominance by *in vitro* assay	BALB/c mice	1.5 × 10^7^ pfu/IP	–	1 × 10^6^*L. major* promastigotes	↑ IFN-γ by CD4^+^ T-cells in spleen	↓ Lesion size; ↓ parasite load in dLN	(45)

CPB	*L. amazonensis*	7 H2 peptides (8–10 aa)	*In silico* prediction (SYFPEITHI)	BALB/c and C57BL/6 mice	30 µg mL^−1^	–	*L. amazonensis* promastigotes	↑ CD8^+^ T-cells proliferation	–	(46)

CPB, CPC, TSA, LeIF, LmSTI, and LPG	*L. major*	18 peptides (9 aa)	*in silico* prediction (SYFPEITHI, BIMAS, EpiJen, RANKpep, Multipred, NetCTL, and nHLApred)	Human PBMC	10 µg mL^−1^	–	–	Peptide pools: ↑ IFN-γ by CD8^+^ T-cells	–	(47)

CPB, CPC, LmSTI, LPG, and other antigens	*L. major*	Polytope (561 bp)	*In silico* prediction (BIMAS)	BALB/c mice	50 μg/SC	–	2 × 10^5^*L. major* promastigotes	↑ IFN-γ by spleen cells	↓ Parasite load in dLN; ↓ footpad swelling	(48)

Phage display library	*Leishmania infantum*	Two phages containing peptides (7 aa)	*In vitro* selection (bio-panning cycles)	BALB/c mice	5 × 10^10^ phages/SC	Saponin	1 × 10^6^*L. amazonensis* promastigotes	↑ IFN-γ, IL-12, and GM-CSF	↓ Parasite load in liver, spleen, dLN, and BM; ↓ lesion size in footpad	(49)

CPA, CPB, GP63, H3 and H4 histone LPG-2	*Leishmania braziliensis*	8 peptides (9 aa)	*In silico* prediction (EPIBOT platform)	BALB/c mice	Peptide-pulsed splenocytes/IV	–	1 × 10^5^*L. braziliensis* promastigotes	Three peptides: ↑ *in vivo* cytotoxicity by specific splenocytes 1	–	(50)

33 different proteins	*L. major*	78 peptides (9 aa)	*In silico* prediction (SYFPEITHI, BIMAS, RANKpep, and NetMHC)	Human PBMC	20 µg (each) or 1 µg per pool	–	–	Six peptides: ↑ granzyme B	–	(51)

Phage display library	*L. major*	6 peptides (6 aa)	*In vitro* selection (bio-panning)	Human PBMC and BALB/c mice	100 μM/SC	–	1 × 10^6^*L. major* metacyclic *promastigotes*	–	P1 and P2 peptides inhibited human monocyte infection. P2: ↓ footpad swelling and ↓ parasite load in footpad, LN, and spleen	(52)

Whole proteome	*L. braziliensis, L. major*, and *L. infantum*	10 peptides (15 aa)	Reverse vaccinology approach	Human PBMC	20 µg mL^−1^	–	–	5 peptides: ↑ PBMC proliferation	–	(53)

*DTH, delay-type hypersensitivity; GP63, glycoprotein 63; KMP-11, kinetoplastid membrane protein-11; CPB, cysteine proteinase B; LACK, Leishmania homolog of receptors for activated C kinase; CPC, cysteine peptidase C; TSA, thiol-specific antioxidant; LeIF, elongation initiation factor-2 alpha subunit; LmsTI, L. major stress-inducible protein 1; LPG, lipophosphoglycan biosynthetic protein; H3 and H4, histones proteins 3 and 4; PBMC, peripheral blood mononuclear cell; APC, antigen-presenting cell; DC, dendritic cell; CPA, cysteine peptidase A; CFA, complete Freund’s adjuvant; IFA, incomplete Freund’s adjuvant*.

Although there is an increase in the number of new vaccines using peptides, a major challenge is how to avoid inactivation or degradation by the immune system and how to enhance the immunogenicity of those peptides. Thus, it is necessary to design vaccines using different approaches and to use other compounds such as adjuvants that can help to enhance the antigen immunogenicity (54).

### Strategies for Peptide Mapping

**Figure 1:**
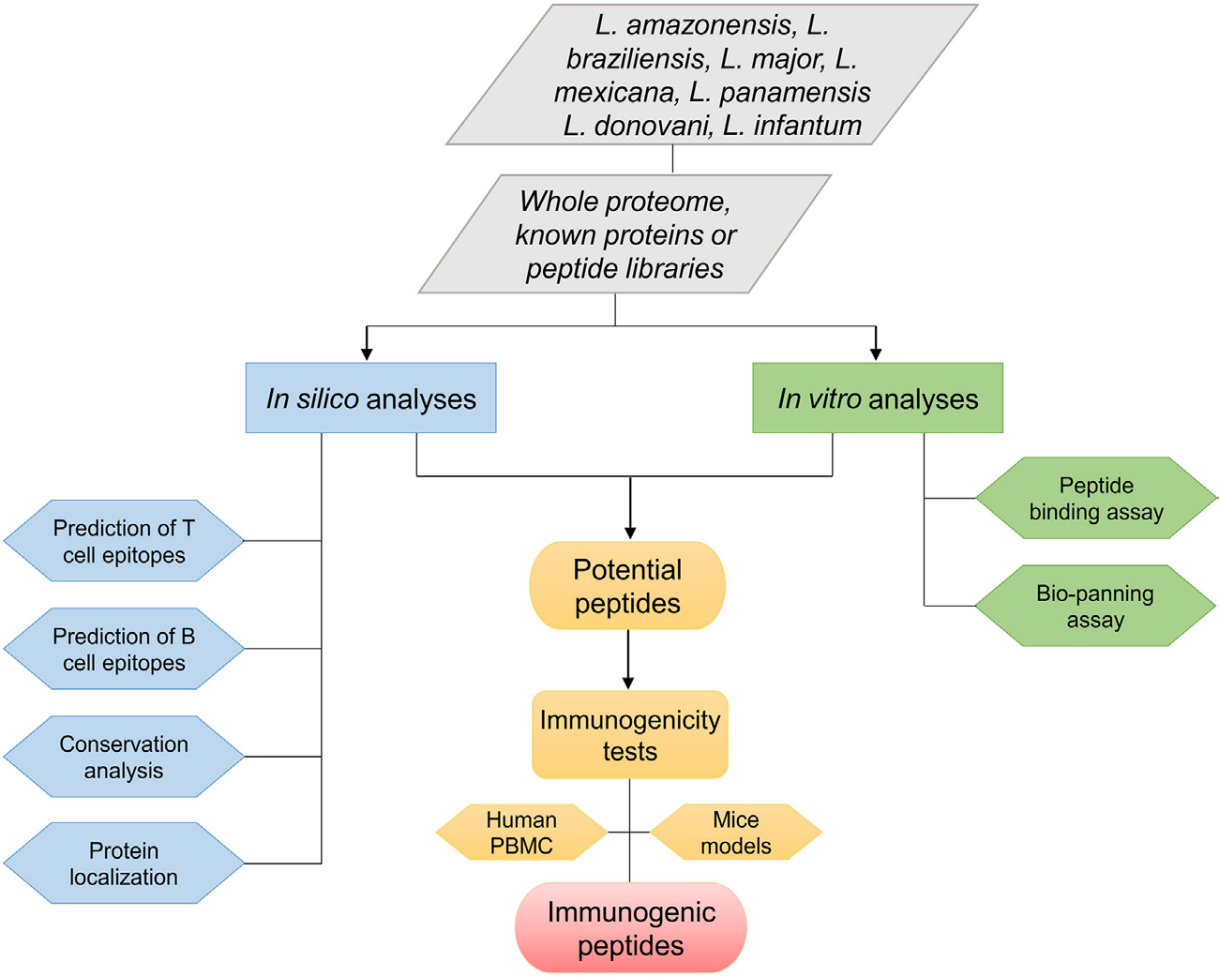
Flowchart of various approaches used to identify promising immunogenic peptides of different *Leishmania* species.

Epitope choice is a crucial stage to develop a peptide vaccine. Consequently, at first, suitable epitopes on the protein or whole proteome of interest need to be mapped. These epitopes must be able to induce strong, long-lasting cellular immunity against *Leishmania* parasites. Peptide epitopes can be identified using various approaches and methodologies (Figure 1). Regarding viscerotropic and dermatotropic species of *Leishmania*, potential immunogenic peptides can be mapped from the whole parasite proteome, proteins that previous elicited immunological outcome, and/or using known peptide libraries. In this scenario, two different analyses can be performed as shown in Figure 1.

The first is the *in silico* approach that is usually focused on prediction of T- and B-cell epitopes, phylogenetic analysis for the identification of conserved leishmanial epitopes *Leishmania* species, or prediction of protein/peptide localization in the parasite. Many algorithms are used to predict affinity binding of peptide epitopes on MHC class I and II molecules, linear or discontinuous B-cell peptide epitopes, or even signal peptides that direct proteins to different subcellular localizations, as shown on Tables 1 and 2. Until recently, vaccine development was associated with conventional methods such as biochemical, immunological, and microbiological approaches using the whole, or part of, the pathogens. With the advent of post-genomic techniques and immunoinformatics for immune system data analysis, reverse vaccinology is becoming a useful tool to design and develop vaccines. Basically, reverse vaccinology uses immunoinformatics for epitope mapping across an entire pathogen genome using predictive algorithms that are able to predict T- and B-cells peptide epitopes. There are many advantages with this strategy, the most important being the decrease of time and cost needed to identify potential vaccine candidates (55, 56). Immunoinformatics started to be applied for leishmaniases research in the early 1990s with T-cell predictive algorithms such as AMPHI method (now it is obsolete) in known immunogenic proteins (35). Various studies have since been performed using peptide epitope predictions in previously known immunogenic proteins. These algorithms have become more powerful in predicting epitopes, and they can be combined to increase the accuracy for large-scale peptide epitope predictions in *Leishmania* proteomes (57, 58). Thus, today it is possible to perform high-throughput screening in whole *Leishmania* proteomes and identify peptides that elicit protective immune responses *in vitro* as well as *in vivo* (43, 53).

The second analysis is *in vitro* toward the discovery of new epitopes to be evaluated in biotechnological applications. Thus, phage display technology is focused on DNA recombination technology, resulting in the expression of a peptide, which mimics the structure of an epitope (termed mimotopes), on the surface of phage clones (59). This approach comprises *in vitro* selection process, binding affinity assays using mimotopes (bio-panning) that mimic peptides exposed on the phage surface with subsequent analysis of these peptides by DNA sequencing based on binding affinity. Phage display has been employed successfully in wide variety of applications, such as vaccine development, drug discovery, diagnosis and therapeutic studies (60). For example, in a study evaluating peptides from *Leishmania infantum* selected throw phage display, the authors observed that two potential peptides (B10-LSFPFPG and C01-FTSFSPY) were able to induce IFN-γ, IL-12, and GM-CSF production in mice splenocytes after challenge. These peptides were able to reduce the parasite load in liver, spleen, lymph nodes, and bone marrow and to reduce lesion size in footpad after *L. infantum* challenge (49). Rhaiem and Houimel (52) showed the potential of these approaches to identify immunogenic peptides. The authors demonstrated that P1 (MSKPKQ) and P2 (MAAKYN) peptides identified by phage display inhibited human monocyte infection by *Leishmania major*, with P2 promoting a reduction in footpad swelling and parasite load in footpad, lymph nodes, and spleen after challenge.

Immunodominance assays and peptide competition assays are used to identify and characterize T-cell epitopes based on antigen-presenting cells (APCs) or fluorescence-labeled peptides (27). In this field, the ability of APCs to present epitopes is assessed using T-cell hybridoma as responder cells. Immunodominant peptides are selected based on their sensitivity and recognition by stablished T-cell lines or hybridoma (61). Finally, it is possible to identify peptide expression of MHC molecules on the surface of APCs, by protein sequencing or immunochemistry. With this approach it is possible to identify leishmanial antigen epitopes for T-cells. For example, Basu et al. (27) identified for the first time a specific T-cell epitopes derived from kinetoplastid membrane protein-11 (KMP-11) protein, and they demonstrated that the use of *in vitro* approaches allows the identification of naturally processed epitopes. In this study mentioned earlier, the authors showed that peptides derived from *Leishmania donovani* promoted a significant IFN-γ production by human CD8^+^ T-cells.

In brief, *in silico* and *in vitro* approaches to map potential peptides seem to be attractive tools for the development of peptide vaccine. The potential peptides identified usually are tested for their immunogenicity capacities using mice models (25, 28–30) or human peripheral blood mononuclear cells (PBMCs) from healthy/non-healthy patients (26, 34, 53) for future peptide vaccine development, as in the studies described in Tables 1 and 2.

### Approaches for Peptide Vaccine Design

**Figure 2:**
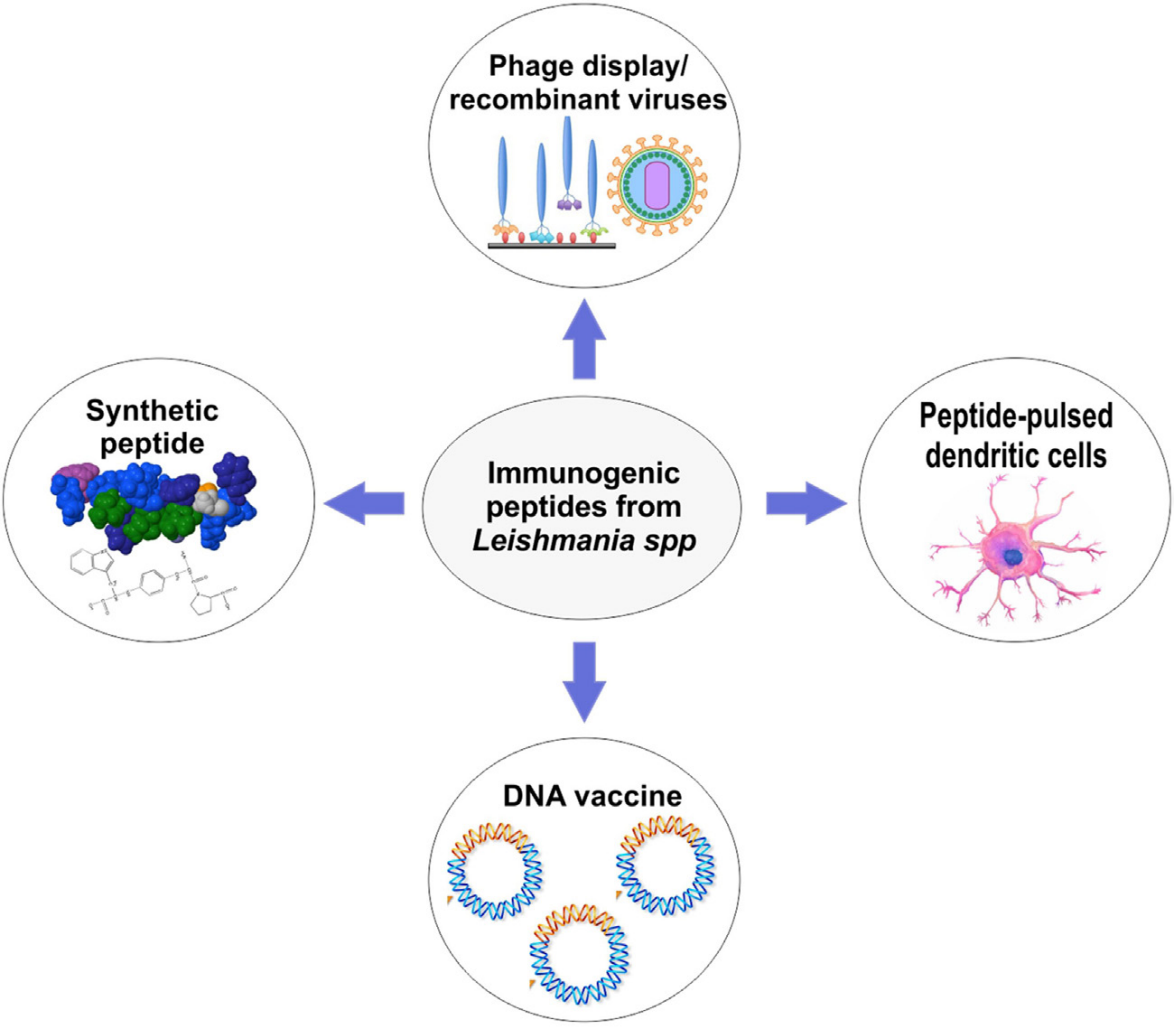
Overview of approaches that can be used for peptide-based vaccines development against cutaneous and visceral leishmaniasis.

Several approaches for peptide-based vaccines design were created to overcome the weak peptide immunogenicity of the peptides and poor delivery (22). In Figure 2, the most widely used approaches to develop peptide-based vaccines are shown. After identification of a potential immunogenic peptide, it can be synthetized and used with a specific adjuvant to solve the issue with low immunogenicity. Nowadays, there is an extensive variety of adjuvants that show efficacy in the induction of immune responses against peptides (54). They are usually agonists of toll-like receptors or proteins on the surface of APCs which recognize pathogen-associated molecular pattern molecules (54). The choice of an adjuvant (or another strategy for peptide vaccine design) is the second major challenge in peptide vaccine development (54). For example, Agallou et al. (29) formulated peptide-based vaccines using an oil–water emulsion, like incomplete Freund’s adjuvant or complete Freund’s adjuvant. The authors demonstrated that peptides selected through *in silico* approaches and associated with Freund’s adjuvants enhanced the immunogenicity of these vaccines; however, peptides derived from the KMP-11 did not display significant immunogenicity. Other adjuvants, for example, surfactants such as poloxamer (8%) (36, 39), or *Quillaja saponaria* bark saponin (31–33) and *Corynebacterium parvum* (35) have been used to compose peptide-based vaccines in the last decades as shown in Tables 1 and 2. Unmethylated CpG oligodeoxynucleotides are also potent agonists for dendritic cells (DCs) activation and maturation, inducing the expression of MHC and costimulatory molecules that play a central role directing Th1 response crucial to *Leishmania* resistance (28).

Another weakness of peptide-based vaccines is the delivery of synthetic peptides. In this context, many approaches must be taken to protect protease-sensitive epitopes from degradation *in vivo* (22). Cellular vaccination, one of these approaches, comprises the *in vitro* stimulation of DCs and subsequent immunization of mice (or another organism). For example, this type of vaccination has shown efficacy in mice challenged with *L. infantum* parasites. Agallou and colleagues, using cellular vaccination, were able to promote a significant IFN-γ and IL-17 production and proliferation of splenocytes. Moreover, a reduction in parasite load in the liver and spleen of challenged mice was observed (28). More recently, with the advance of recombinant DNA technology, DNA sequences for peptides have been used to design DNA vaccines (25, 48) or to develop attenuated virus (e.g., adenovirus) or phage display that can translate the peptide sequence and drive strong T-cell responses (32, 45, 49). DNA and adenovirus vaccines (Figure 2) have demonstrated to be strong inducers of T-cell activation leading to intracellular parasite control (24, 30). Several studies emphasize that these approaches are the future for peptide vaccine design (24, 25, 30, 48). For example, Das et al. (24) proposed a polyepitope DNA vaccine that showed promising results in mice. The DNA vaccine elicited strong immunogenicity and promoted parasite load reduction in liver and spleen.

To test those vaccines for cutaneous and visceral leishmaniases, BALB/c mice are frequently used for immunological studies. The use of peptides in vaccine composition seems to be promising and has shown interesting results regarding the immune response (Tables 1 and 2). Thus, peptides can trigger important markers related to protection against *Leishmania* sp., such as the development of a strong cellular response by delay-type hypersensitivity, production of important Th1 cytokines (mainly IFN-γ), and *in vitro* proliferation of specific T-cells. Regarding vaccine potency and efficacy, the studies show that different peptide vaccine approaches can promote reduction of lesion size and parasite load in affected organs such as liver, spleen, bone marrow, and lymph node (Tables 1 and 2). These emphasize the promising field of using peptides in vaccine development for different diseases including leishmaniases (9, 62, 63). Despite the promising field and novelty of peptide-based vaccines, to date there are not approved peptide vaccine for human or animal use (64).

## Vaccine Peptides: New Perspectives

### Chimeric and Polypeptide Vaccines

Some research groups aiming to develop *Leishmania* vaccines to prevent the visceral or cutaneous forms of leishmaniases have tested polyproteins containing multiple antigenic epitopes from *Leishmania* associated with different adjuvants (65, 66). The polyprotein KSAC (a fusion protein composed of KMP-11, SMT, A2, and cysteine peptidase B) was shown to be protective in BALB/c mice against early lesion development after sand fly challenge with *L. major*, with an approximately 50-fold reduction in parasite burden after 5 weeks (67). Alves-Silva et al. (68) showed that a chimeric protein containing CD4^+^ and CD8^+^ T-cell epitopes for *L. donovani* nucleoside hydrolase 36 promotes cross-protection against *Leishmania amazonensis* challenge in a BALB/c mice model. In a similar study, Martins et al. (69) demonstrated that a chimeric protein, displaying murine and human MHC class I- and II-specific epitopes from four proteins (LiHyp1, LiHyp6, LiHyV, and HRF) identified in an immunoproteomic study of visceral leishmaniasis antigens, was protective against heterologous challenge with *L. amazonensis* in a murine model. All these vaccines demonstrated strong potential to be used in phase I clinical trials. However, it is important to combine these antigens with approved adjuvants for safe use in humans, although recombinant *Leishmania* polyproteins, chimeric, and/or polypeptide vaccines show good manufacturing practices and regulatory approval. These studies show the potential of engineering chimeric peptide/protein vaccines.

### Nanovaccines

Recently, nanotechnology has gained attention in vaccine development, as it provides a path for a promising antigen delivery system that can both stabilize vaccine antigens and act as adjuvants. This approach has been intensively implemented in the therapeutic treatment of cancer and infectious diseases (70–72). Nanovaccines consist of nanoscale particles attached or formulated with antigenic components to drive a specific desired immune response (73). The nanoparticles protect the encapsulated antigenic molecules from degradation by allowing sustained release that maximizes exposure to the immune system, by site-specific delivery, and by enhancing the bioavailability of antigens (73–76). Several studies have demonstrated that peptide-based vaccines may benefit from particulate delivery systems that mimic the size and structure of a pathogen, which favors uptake by DCs and enhances the probability of peptide cross-presentation (77–79). Athanasiou et al. (75) observed that chimeric peptide vaccines containing HLA-restricted epitopes of three immunogenic *L. infantum* proteins (cysteine peptidase A, histone H1, and KMP-11) encapsulated in poly(lactic-co-glycolic) acid nanoparticles with the adjuvant monophosphoryl lipid A induced IL-12 production, promoted allogeneic T-cell proliferation and intracellular production of IFN-γ by CD4^+^ and CD8^+^ T-cell subsets, and thus stimulated significant protection against *L. infantum* infection. Thus, a suitable biocompatible delivery system with the appropriate adjuvant is an improved approach for the development of a vaccine against several diseases, including visceral leishmaniasis.

## Expert Commentary and Conclusion

The leishmaniases have shown an impressive capacity to spread around the world, disclosing a scarcity of effective management of the epidemiology. Although it is already endemic in many continents, due to global warming, these parasitic diseases may spread into new geographic areas. Thus, in our opinion, the best way to prevent this epidemiological scenario is prophylaxis using vaccine immunizations. Therefore, researchers need to discover novel approaches for identifying promising antigens for vaccine development against leishmaniases. “Rational vaccinology” is a driving force in the discovery of specific epitopes to enhance the immune response and capacity of specific immune cells to eliminate *Leishmania* parasites.

In this review, we note that a solution for this problem could be achieved with the development of new approaches to identify potential immunogenic peptides. We believe that the most promising approach is immunoinformatics, which can be used for computational mining of proteomic/genomic databases of *Leishmania*. This approach allows for faster and more cost-efficient peptide vaccine development. The selection of peptides as antigens may provide a safer solution for leishmaniases, as they are able to promote a specific immune response, show better stability, and can be produced at a more reasonable cost, when compared with whole parasite or recombinant protein antigens. One weakness, however, is that they are liable to immune barriers. To prevent this possible problem, researchers must identify new vaccine designs based on different formulation strategies to define which ones can produce a desired immune response. Indeed, many studies have focused on vaccine design methodologies, making it possible to create mechanisms to deliver the entire peptide into the immune system (mainly APCs) and thus promoting enhanced peptide immunogenicity. Recently developed techniques for vaccine design, such as polypeptides, chimeric vaccines, the use of adjuvants, cellular vaccination, and nanovaccines, which allow for a combination of nanoparticles and specific peptides, seem to be the future of vaccine development and hopefully will lead to a safe and effective vaccine against leishmaniases.

## Statement

The authors gathered the background information through an extensive literature search relevant to the topic of interest. The first step was to select original research that is described in Tables 1 and 2. In this concern, the authors consulted PubMed database (https://www.ncbi.nlm.nih.gov/pubmed/) to identify critical articles and to track down “landmark” articles. For that, Boolean operators were used to combine search terms in PubMed as follows: “*Leishmania* AND synthetic vaccine,” “Peptide vaccine AND *Leishmania*,” and “Epitope vaccine AND *Leishmania*” considering a period (since 1990–2017) trying to offer a retrospective of vaccine design based on leishmanial peptides. The searches outcomes were manually curated to exclude review articles and those not versing about peptides. Furthermore, we excluded manuscripts addressing the use of peptides for leishmaniasis serodiagnosis. From the remaining articles, we selected 30 full-text articles covering almost all the *Leishmania* species that cause cutaneous and visceral leishmaniases. These articles comprise the use (selection and testing) of peptides for *Leishmania* vaccine design and development. They address different approaches to peptide selection, the use of various methods to design peptide-based vaccines and the use of different experimental models (e.g., mice or PBMC from infected patients) to screen and evaluate the efficacy of immunogenic peptides. Moreover, the articles emphasize the classical markers related to immune system activation (e.g., IFN-γ production, proliferation of T-lymphocytes after *in vitro* stimulus) and the capacity of these peptides to reduce the parasite load and lesion in affected organs which are important aspects for a vaccine design to be considered successful.

## Author Contributions

All the authors participated with suggestions and the development of this manuscript; RB participated in the selection of studies related to peptide vaccines development against leishmaniasis in the literature. RB, JC, LR, JV, FM, BR, and RA-S participated in drafting the article and/or revising it critically for important intellectual content, and also created the figures and tables. JR, DR, and AR participated in the study conception, critical revision of the article, and supervision.

## Conflict of Interest Statement

The authors declare that the research was conducted in the absence of any commercial or financial relationships that could be construed as a potential conflict of interest.
